# Characterization of cases and epidemiological and operational indicators of leprosy: analysis of time series and spatial distribution, Piauí state, Brazil, 2007-2021

**DOI:** 10.1590/S2237-96222024v33e2023090.en

**Published:** 2024-01-15

**Authors:** Ian da Costa Araújo Barros, Carliane da Conceição Machado Sousa, Neylany Raquel Ferreira da Silva, Márcio Dênis Medeiros Mascarenhas

**Affiliations:** 1 Universidade Federal do Piauí, Curso de Graduação em Medicina, Teresina, PI, Brazil; 2 Universidade Federal do Piauí, Programa de Pós-graduação em Saúde e Comunidade, Teresina, PI, Brazil; 3 Universidade Federal do Piauí, Centro de Inteligência em Agravos Tropicais Emergentes e Negligenciados, Teresina, PI, Brasil

**Keywords:** Leprosy, Disease Notification, Health Information Systems, Time Series Studies, Spatial Distribution, Lepra, Notificación de Enfermedades, Sistemas de Información en Salud, Estudios de Series Temporales, Distribución Espacial, Hanseníase, Notificação de Doenças, Sistemas de Informação em Saúde, Estudos de Séries Temporais, Distribuição Espacial

## Abstract

**Objective:**

To analyze epidemiological characteristics, temporal trends and spatial distribution of leprosy cases and indicators in the state of Piauí, 2007-2021.

**Methods:**

This was an ecological time-series study using data from the Notifiable Health Conditions Information System, describing the spatial distribution and the temporal trend of leprosy using Prais-Winsten regression.

**Results:**

A total of 17,075 new cases of leprosy were reported. There was a falling trend in the overall detection rate [annual percentage change (APC) = -6.3; 95%CI -8.1;-4.5)], detection in children under 15 years of age (APC = -8,6; 95%CI -12,7;-4,3) and detection of cases with grade 2 physical disability (APC = -4,4; 95%CI -7,0;-1,8). There was a rising trend in the proportion of multibacillary cases. Spatial distribution of the average detection rate identified hyperendemic areas in the Carnaubais, Entre Rios, Vale dos Rios Piauí e Itaueiras regions.

**Conclusion:**

High leprosy detection rates were found, despite the falling trend of indicators, except the proportion of multibacillary cases.

## Study contributions

**Main results:**

Between 2007 and 2021, Piauí showed an improving trend in all indicators analyzed, except the proportion of new multibacillary cases. Spatial distribution identified hyperendemic areas in different regions of the state.

**Implications for services:**

This study provides health services with a broad overview of the epidemiological situation of leprosy in Piauí, with evidence of the populations and locations most affected by the disease, whereby public authorities need to reinforce the promotion of health actions.

**Perspectives:**

It is expected that actions to prevent and control the disease will be adopted, such as offering early diagnosis, ensuring correct treatment and implementing measures to prevent physical disabilities.

## RESUMEN

**Objetivo:**

Analizar características epidemiológicas, tendencias temporales, distribución espacial de casos y indicadores de lepra en el estado de Piauí, 2007-2021.

**Métodos:**

Estudio ecológico de series temporales con datos del Sistema de Información de Enfermedades de Notificación que describen la distribución espacial y la tendencia temporal de la lepra mediante la regresión de Prais-Winsten.

**Resultados:**

Se notificaron 17.075 nuevos casos de lepra. Hubo tendencia decreciente en las tasas de detección general [variación porcentual anual (VPA) = -6,3; IC_95_% -8,1; -4,5], detección en menores de 15 años (VPA = -8,6; IC_95_% -12,7; -4,3), y detección de casos con discapacidad física grado 2 (VPA = -4,4; IC_95_%-7,0; -1,8). Hubo una tendencia creciente en la proporción de casos multibacilares. La distribución espacial de la tasa promedio de detección identificó áreas hiperendémicas en las regiones de Carnaubais, Entre Rios, Vale dos Rios Piauí y Itaueiras.

**Conclusión:**

Se observaron altas tasas de detección de lepra, a pesar de la tendencia decreciente de los indicadores, excepto la proporción de casos multibacilares

## RESUMO

**Objetivo:**

Analisar características epidemiológicas, tendência temporal, distribuição espacial de casos e indicadores da hanseníase no estado do Piauí, 2007-2021.

**Métodos:**

Estudo ecológico de séries temporais com dados do Sistema de Informação de Agravos de Notificação, descrevendo a distribuição espacial e a tendência temporal da hanseníase pela regressão de Prais-Winsten.

**Resultados:**

Foram notificados 17.075 novos casos de hanseníase. A distribuição espacial da taxa média de detecção identificou áreas hiperendêmicas nas regiões Carnaubais, Entre Rios, Vale dos Rios Piauí e Itaueiras. Verificou-se tendência decrescente nas taxas de detecção geral [variação percentual anual (VPA) = -6,3; IC_95_% -8,1;-4,5], de detecção em menores de 15 anos (VPA = -8,6; IC_95_%12,7;-4,3) e de detecção de casos com grau 2 de incapacidade física (VPA = -4,4; IC_95_%-7,0;1,8), e tendência crescente na proporção de casos multibacilares.

**Conclusão:**

Observaram-se elevadas taxas de detecção de hanseníase, apesar da tendência decrescente dos indicadores, exceto a proporção de casos multibacilares.

## INTRODUCTION

Despite the significant reduction in the burden of leprosy after the introduction of multidrug therapy (MDT), the disease persists as a public health problem, especially in underdeveloped nations. It is endemic in tropical regions, such as Brazil, and is considered one of the most important neglected diseases. Globally, the World Health Organization (WHO) reported 140,594 new leprosy cases in 2021, with the majority being found in India, Brazil and Indonesia.^1^

In Brazil, leprosy has been a challenge for decades and the country occupies second place in the global ranking of countries with a high burden of the disease.^1^ According to the Brazilian Ministry of Health, the country reported 18,143 new leprosy cases in 2021, with a detection rate of 8.51 cases per 100,000 inhabitants. The state of Piauí, located in the Northeast region of Brazil, accounted for 652 cases in the same year, with a detection rate of 19.82 cases per 100,000 inhabitants, the fifth highest rate among the Brazilian Federative Units.^2^

The COVID-19 pandemic posed new challenges emerged for healthcare systems, compromising access to public healthcare services and the priority given to emergency cases. Active surveillance and diagnosis of leprosy were often interrupted or became impossible due to social distancing measures and restricted access to health services.^3,4^

Considering the reality of Piauí, with significant vulnerability of the population in situations of poverty and structural problems in health care networks, the state stands out for its high incidence of neglected tropical diseases (NTDs) and high associated mortality rates.^5^ Leprosy generates hospital costs in terms of inpatient stay, treatment and rehabilitation, in addition to generating considerable morbidity due to physical disabilities and deformities, leading to greater marginalization, stigma and prejudice towards people with the disease. Limited access of the most vulnerable populations to health education provides a favorable environment for transmission.^6^

Given the relative scarcity of studies on leprosy in Piauí in a period that encompasses the COVID-19 pandemic, it is essential to have information on the current temporal and spatial patterns of the disease in the state, so that possible demands for health surveillance, prevention, treatment and rehabilitation actions in the Brazilian National Health System (*Sistema Único de Saúde* - SUS) can be identified in priority regions. This therefore study aimed to describe the epidemiological characteristics, temporal trend and spatial distribution of cases, as well as to analyze leprosy indicators in the state of Piauí, Brazil, from 2007 to 2021.

## METHODS

### Study design and period 

**Figure 1:**
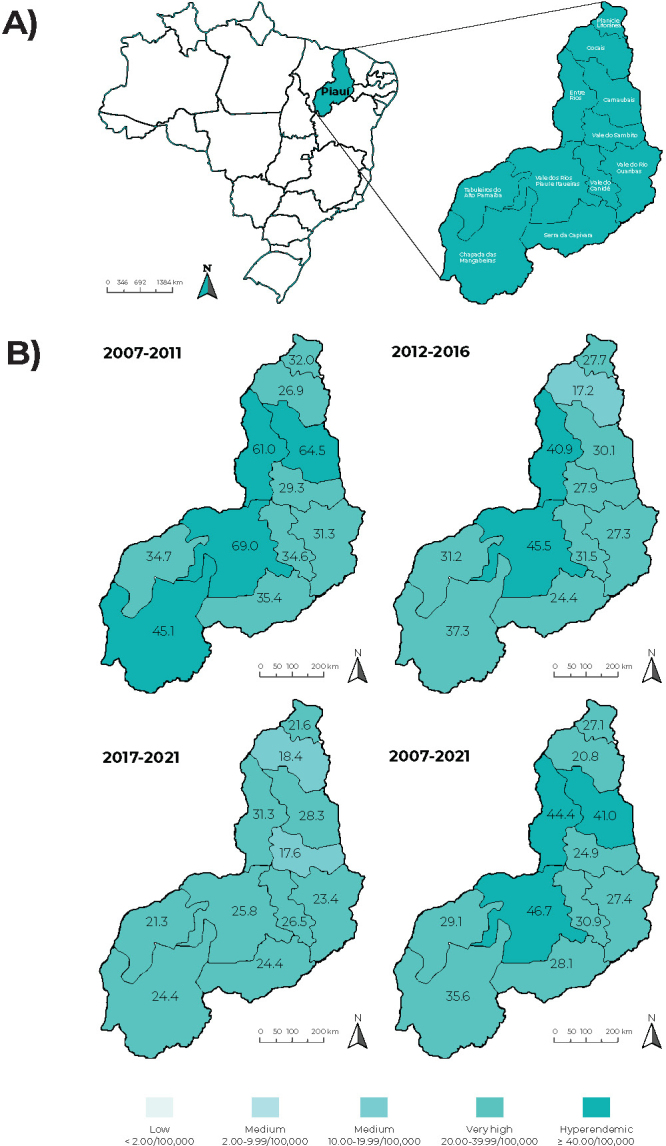
Health regions of the state of Piauí, in the Northeast of Brazil (A), and spatial distribution of new leprosy case detection rates by health regions of residence (B), Piauí, Brazil, 2007-2021

This was an epidemiological, observational, ecological analytical time-series study, using records of leprosy cases resident in the Brazilian state of Piauí, diagnosed between 2007 and 2021. The units of analysis were Piauí’s 11 health regions and its five health macro-regions (Figure 1A).

### Study location 

In 2021 the estimated population of the state of Piauí was 3,289,290 inhabitants, with demographic density of 12.4 inhab. per km². With 251,755.481 km², it corresponds to the 11^th^ largest Brazilian state in terms of its territorial area. In 2010, Piauí was the Brazilian state with the fourth lowest Human Development Index (HDI), below the country’s average HDI of 0.765.^7^

### Data source 

Data on people diagnosed with leprosy are recorded by health professionals on individual investigation forms, with subsequent input to the Notifiable Health Conditions Information System (*Sistema de Informação de Agravos de Notificação* - SINAN) to form the national database. The database, with anonymous data, can be accessed via the website of the Brazilian National Health System SUS Information Technology Department (*Departamento de Informática do Sistema Único de Saúde* - DATASUS), under the responsibility of the Ministry of Health. Data referring to the population resident in Piauí were extracted from projections made by the Brazilian Institute of Geography and Statistics (*Instituto Brasileiro de Geografia e Estatística* - IBGE), retrieved from the DATASUS website by means of the TabNet tabulator.^6^ All data used in this study were obtained on May 11, 2023, from the DATASUS website.^2^

### Study variables 

Leprosy cases were described according to sociodemographic and clinical variable aggregates. The sociodemographic variable aggregates were: sex (male, female), age group (in years: 0-14, 15-39, 40-59, 60 or over), self-reported race/skin color (White, Black, Asian, mixed race, Indigenous, no information), health region of residence (Carnaubais, Chapada das Mangabeiras, Cocais, Entre Rios, Planície Litorânea, Serra da Capivara, Tabuleiros do Alto Parnaíba, Vale do Canindé, Vale do Rio Guaribas, Vale do Sambito, Vale dos Rios Piauí and Itaueiras) and health macro-region of residence (Litoral, Meio-Norte, Semiárido, Cerrados). The clinical variables were: operational classification (paucibacillary – PB, multibacillary – MB, no information); grade of disability (GD) at diagnosis (grade 0 – G0D, grade 1 – G1D, grade 2 – G2D, no information); and clinical form (indeterminate, tuberculoid, borderline, lepromatous, no information).

### Epidemiological and operational indicators 

The indicators were calculated following the definitions of purpose, calculation method and interpretation parameters recommended by the Ministry of Health:^8^

a) New case detection rate per 100,000 inhabitants (total and stratified according to sociodemographic variables):
  Purpose: to determine leprosy morbidity strength, magnitude and trend over time;
  Calculation method: number of new cases residing in a given location and diagnosed in the year of assessment divided by the total population in the same location and period, multiplied by 100,000;
  Parameters: low (< 2/100,000), medium (29.99/100,000), high (10-19.99/100,000), very high (20-39.99/100,000), hyperendemic (≥ 40/100,000).
b) New case detection rate in the population aged zero to 14 years per 100,000 inhabitants:
  Purpose: to measure the strength of recent transmission of the endemic and its trend;
  Calculation method: number of new cases in children under 15 years old living in a given location and diagnosed in the year of assessment divided by the population aged zero to up to 14 years old in the same location and period, multiplied by 100,000;
  Parameters: low (< 0.50/100,000), medium (0.50-2.49/100,000), high (2.50-4.99/100,000), very high (5.00-9.99/100,000), hyperendemic (≥ 10.00/100,000).
c) New case detection rate with G2D per 100,000 inhabitants:
  Purpose: to assess disabilities caused by leprosy in the general population;
  Calculation method: number of new cases with G2D, residing in a given location and diagnosed in the year of assessment divided by the population residing in the same location and period, multiplied by 100,000;
  Parameters: there are no established parameters.
d) Proportion of cases according to operational classification:
  Purpose: to assess the risk of developing complications, as well as the correct level of MDT;
  Calculation method: number of new multibacillary cases residing in a given location and diagnosed in the year of assessment, divided by the total number of new cases of leprosy residing in a given location and diagnosed in the year of assessment, multiplied by 100;
  Parameters: there are no established parameters.

### Statistical analysis 

Initially, data relating to leprosy case characteristics were analyzed using descriptive statistics of the absolute and relative frequencies of the selected variables. Next, the new case detection rates were calculated for each year and the average detection rate was calculated by dividing the average number of new leprosy cases per year by the resident population of the middle year (2014), and multiplying the result by 100,000 inhabitants. The proportion of multibacillary and paucibacillary cases was calculated by dividing the number of cases in each operational category by the total number of new leprosy cases, multiplied by 100 inhabitants. 95% confidence intervals (95%CI) were calculated for the detection rates. Magnitude of association between the explanatory variables and the detection of new leprosy cases was determined by calculating the detection rate ratio (DRR) and respective 95%CIs, whereby statistical differences were verified by Pearson’s chi-square test or Fisher’s exact test (when there were values ​​lower than 5) taking a 5% significance level (p-value < 0.05). 

The temporal trend of the indicators was analyzed using Prais-Winsten linear regression, which considers serial autocorrelation.^9^ To this end, regression analysis of the decimal logarithm (log base 10) of each indicator (dependent variable – Y) according to year of diagnosis (independent variable – X) was performed, considering the formula:


Log(Yt)=β0+β1x,

where: Log(Yt): value of the decimal logarithm of the indicator Y in year t; β0: constant or intercept; β1: linear trend coefficient; x: year of diagnosis. 

With the values ​​of the β1 coefficient and the standard error (EP) obtained through Prais-Winsten linear regression analysis, annual percentage change (APC) and respective 95%CIs were calculated using the formulas:


$$\mathrm{APC}=(-1+10^{\beta 1})x100;\;\text{and,}$$

$$95\text{\%CI}=(-1+10^{\text{β1 minimum}})x100;(-1+10^{\text{β1 maximum}})x100.$$

Trends were classified as rising (when the β1 coefficient was positive and p-value < 0.05 using the Wald test), falling (when the β1 coefficient was negative and p-value < 0.05 using the Wald test) or stationary (Wald test p-value > 0.05, regardless of the value of the β1 coefficient).

The average new case detection rate was calculated for each health region by five-year periods (2007-2011, 2012-2016, 2017-2021) and for the total period (2007-2021). The results were presented on maps of the territory of Piauí, divided into 11 health regions. Rate averages were classified according to magnitude patterns (low, medium, high, very high, hyperendemic), in accordance with the interpretation parameters recommended by the Ministry of Health.^8^

The data obtained from TabNet were exported to Microsoft® Excel®, where absolute and relative distributions, epidemiological and operational indicators and decimal logarithm values ​​were calculated, in addition to creating graphs. Then, the values ​​of the decimal logarithms of the indicators underwent Prais-Winsten linear regression analysis using Stata version 14 (StataCorp LP, College Station EUA). The TabWin program was used to build the maps.

### Ethical aspects 

This study was conducted with publicly accessible secondary data, guaranteeing the confidentiality and anonymity of all participants whose records were analyzed, in accordance with the recommendations of National Health Council Resolution No. 466, dated December 12, 2012, so that submission to a Research Ethics Committee was not necessary.

## RESULTS

A total of 17,075 new leprosy cases were reported in Piauí between 2007 and 2021. There was a higher proportion among the male population (53.3%; n = 9,095), in the 40-59 age group (34.6%; n = 5,906), those of mixed race/skin color (67.9%; n = 11,592), residents of the Entre Rios health region (46.9%; n = 8,007) and the Meio-Norte health macro-region (52.7%; n = 8,999) (Table 1). Regarding clinical aspects, there was a higher proportion of multibacillary cases (58.4%; n = 9,978), grade 0 disability at the time of diagnosis (65.5%; n = 11,190) and borderline clinical form (32.3%; n = 5,509) (Table 2). 

**Table 1:** Number, proportion and detection rate of leprosy cases (per 100,000 inhabitants) according to sociodemographic variables, Piauí, Brazil, 2007-2021

Variables	Cases			Detection rate^a^ (per 100,000)		DRR^c^	95%CI^b^	p-value^d^
	**N**	**%**		**Rate**	**95%CI** ^b^			
**Total**	**17,075**	**100.0**		**36.5**	**34.5;38.6**	**-**	**-**	**-**
**Sex**								
Female	7,980	46.7		32.2	29.6;35.8	1.00	-	-
Male	9,095	53.3		40.9	37.9;44.2	1.27	1.13;1.43	< 0.001
**Age group (in years)**								
≤ 14	1,188	7.0		10.7	8.6;13.2	1.00	-	-
15-39	5,744	33.6		31.9	29.0;35.0	2.99	2.38;3.77	< 0.001
40-59	5,906	34.6		57.8	52.4;63.8	5.43	4.30;6.85	< 0.001
≥ 60	4,237	24.8		71.2	63.0;80.4	6.68	5.24;8.53	< 0.001
**Race skin/color**								
White	2,313	13.5		18.2	15.4;21.5	1.00	-	-
Black	2,563	15.0		57.7	49.6;67.1	3.17	2.54;3.98	< 0.001
Asian	230	1.3		22.5	13.3;37.4	1.24	0.73;2.11	0.217
Mixed race	11,592	67.9		41.5	38.8;44.5	2.29	1.91;2.74	< 0.001
Indigenous	59	0.3		67.9	1.4;264.5	3.74	0.93; 15.09	0.103^e^
No information	318	1.9		-		-	-	-
**Health region of residence**								
Carnaubais	992	5.8		19.7	13.9;28.0	1.14	0.73;1.78	0.289
Chapada das Mangabeiras	1,031	6.0		45.8	37.2;56.4	2.63	1.85;3.74	< 0.001
Cocais	1,237	7.2		17.9	14.1;22.6	1.03	0.71;1.48	0.443
Entre Rios	8,007	46.9		47.9	44.1;51.9	2.75	2.05;3.69	< 0.001
Planície Litorânea	1,117	6.5		17.4	13.1;23.1	1.00	-	-
Serra da Capivara	612	3.6		27.4	20.0;37.4	1.57	1.04;2.39	0.016
Tabuleiros do Alto Parnaíba	202	1.2		34.2	20.6;56.1	1.97	1.12;3.47	0.008
Vale do Canindé	498	2.9		37.1	27.1;50.6	2.13	1.40;3.24	< 0.001
Vale do Rio Guaribas	1,523	8.9		32.5	27.2;38.9	1.87	1.34;2.61	< 0.001
Vale do Sambito	399	2.3		34.7	25.0;47.9	1.99	1.30;3.06	< 0.001
Vale dos Rios Piauí and Itaueiras	1,457	8.5		49.9	41.2;60.5	2.87	2.04;4.04	< 0.001
**Health macro-region of residence**								
Litoral	2,354	13.8		17.7	14.8;21.2	1.00	-	-
Meio-Norte	8,999	52.7		44.5	41.2;48.2	2.52	2.07;3.07	< 0.001
Semiárido	2,420	14.2		33.7	29.4;38.8	1.91	1.52;2.34	< 0.001
Cerrados	3,302	19.3		41.8	36.9;47.4	2.37	1.90;2.94	< 0.001

a) Average detection rate: Average number of new cases per year of diagnosis, divided by the resident population in the middle year (2014), multiplied by 100,000 inhabitants; b) 95%CI: 95% confidence interval; c) DRR: Detection rate ratio; d) Pearson’s chi-square test; e) Fisher’s exact test.

**Table 2:** Number, proportion and detection rate of leprosy cases (per 100,000 inhabitants) according to clinical variables, Piauí, Brazil, 2007-2021

Variables	Cases			Detection rate^a^ (per 100,000)		DRR^c^	95%CI^b^	p-value^d^
	**N**	**%**		**Rate**	**95%CI** ^b^			
**Total**	**17,075**	**100.0**		**36.5**	**34.5;38.6**	**-**	**-**	**-**
**Operational classification** ^e^								
Paucibacillary	7,096	41.6		14.0	12.8;15.4	1.00	-	-
Multibacillary	9,978	58.4		22.5	20.9;24.2	1.61	1.43;1.81	< 0.001
**Grade of disability**								
Grade 0	11,190	65.5		23.6	22.0;25.3	11.55	8.98;14.85	< 0.001
Grade 1	3,251	19.0		6.3	5.5;7.2	3.09	2.34;4.08	< 0.001
Grade 2	956	5.6		2.0	1.6;2.6	1.00	-	-
Not assessed	1,145	6.7		-		-	-	-
No information	533	3.1		-		-	-	-
**Clinical form**								
Indeterminate	3,913	22.9		7.5	6.6;8.5	1.42	1.16;1.73	< 0.001
Tuberculoid	2,709	15.9		5.3	4.5;6.1	1.00	-	-
Borderline	5,509	32.3		11.5	10.4;12.8	2.19	1.83;2.62	< 0.001
Lepromatous	2,561	15.0		6.2	5.4;7.1	1.18	0.97;1.45	0.054
Not classified	1,799	10.5		-		-	-	-
No information	584	3.4		-		-	-	-

a) Average detection rate: Average number of new cases per year of diagnosis, divided by the resident population in the middle year (2014), multiplied by 100,000 inhabitants; b) 95%CI: 95% confidence interval; c) DRR: Detection rate ratio; d) Pearson’s chi-square test; e) One record with no information was excluded.

The average annual detection rate of new leprosy cases in the general population was 36.5/100,000 inhab. (95%CI 34.5;38.6), ranging from 46.8/100,000 in 2007 to 19.8/100,000 in 2021. The annual detection rate of new leprosy cases was significantly higher in the male population (40,9/100,000 inhab.), among people aged 60 or over (71.2/100,000 inhab.), those who reported themselves as being Black (57.7/100,000 inhab.) and of mixed race (41.5/100,000 inhab.) and among residents of the Vale dos Rios Piauí and Itaueiras (49.9/100,000 inhab.) and Entre Rios (47.9/100,000 inhab.) regions, in addition to the Meio-Norte macro-region (44.5/100,000 inhab.) and the Cerrados macro-region (41.8/100,000 inhab.) (Table 1). The detection rate was significantly higher for multibacillary cases (22.5/100,000 inhab.), grade 0 disability (23.6/100,000 inhab.) and borderline (11.5/100,000 inhab.) and indeterminate clinical forms (7.5/100,000 inhab.) (Table 2).

**Figure 2:**
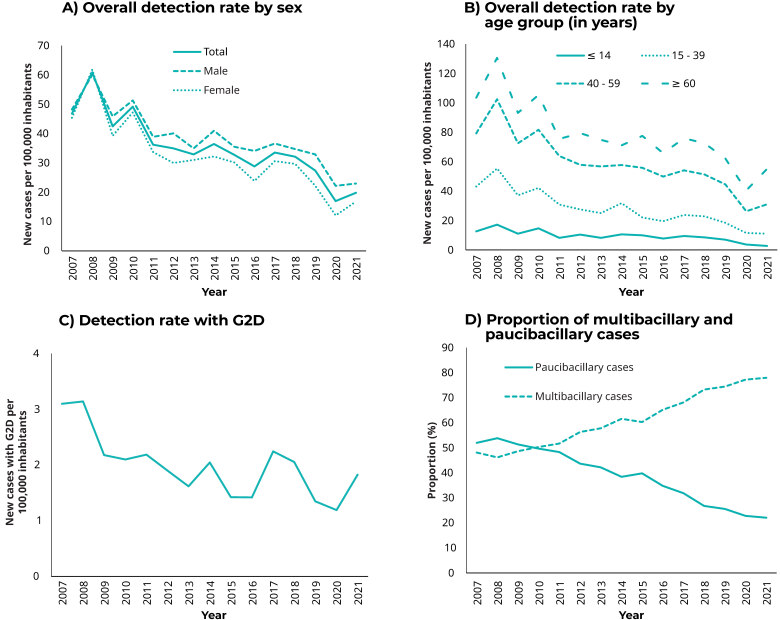
Evolution of leprosy epidemiological and operational indicators, Piauí, Brazil 2007-2021

There was a falling trend in the annual detection rate of new leprosy cases in Piauí (APC = -6.3; 95%CI -8.1;-4.5). The greatest reductions were found in females (APC = -7.6; 95%CI -9.9; -5.3), people aged 15 to 39 years old (APC = -9.0; 95%CI -11.1;-6.9) and under 15 years of age (APC = -8.6; 95%CI -12.7;-4.3) and in residents of the Entre Rios region (APC = -7.4; 95%CI -9.7;-5.1) and the Vale dos Rios Piauí and Itaueiras region (APC = -9.2; 95%CI -10.8;-7.6). Only the Vale do Rio Guaribas region showed stability in the new leprosy case detection rate. The detection rate of cases with grade 2 disability at diagnosis showed a falling trend (APC = -4.4; 95%CI 7.0;1.8). On the other hand, the proportion of new multibacillary cases showed a rising trend (APC = 4.1; 95%CI 3.8;4.5) (Table 3, Figure 2)

a) G2D: Grade 2 disability at the time of diagnosis.

**Table 3:** Trend of leprosy epidemiological and operational indicators, Piauí, Brazil, 2007-2021

Indicators	Average	APC^b^ (95%CI)^c^	p-value^d^	Trend
**new case detection rate (per 100,000 inhabitants)**				
**Total**	**36.5**	**-6.3 (-8.1;-4.5)**	**< 0.001**	**Falling**
Female	32.2	-7.6 (-9.9;-5.3)	< 0.001	Falling
Male	40.9	-5.2 (-6.6;-3.7)	< 0.001	Falling
**Age group (in years)**				
≤ 14	10.7	-8.6 (-12.7;-4.3)	0.001	Falling
15-39	31.9	-9.0 (-11.1;-6.9)	< 0.001	Falling
40-59	57.8	-6.8 (-8.7;-4.9)	< 0.001	Falling
≥ 60	71.2	-5.4 (-6.9;-3.8)	< 0.001	Falling
**Health region of residence**				
Carnaubais	19.7	-6.5 (-11.0;-1.7)	0.013	Falling
Chapada das Mangabeiras	45.8	-6.8 (-9.7;-3.9)	< 0.001	Falling
Cocais	17.9	-4.2 (-7.7;-.05)	0.028	Falling
Entre Rios	47.9	-7.4 (-9.7;-5.1)	< 0.001	Falling
Planície Litorânea	17.4	-3.7 (-6.4;-0.8)	0.015	Falling
Serra da Capivara	27.4	-3.6 (-5.8;-1.4)	0.004	Falling
Tabuleiros do Alto Parnaíba	34.2	-3.9 (-7.0;-0.6)	0.023	Falling
Vale do Canindé	37.1	-3.9 (-7.4;-0.4)	0.033	Falling
Vale do Rio Guaribas	32.5	-3.1 (-6.1;0.1)	0.055	Stationary
Vale do Sambito	34.7	-4.6 (-8.6;-0.4)	0.033	Falling
Vale dos Rios Piauí e Itaueiras	49.9	-9.2 (-10.8;-7.6)	< 0.001	Falling
**Health macro-region of residence**				
Litoral	17.7	-3.8 (-6.3;-1.3)	0.006	Falling
Meio-Norte	44.5	-7.4 (-9.5;-5.3)	< 0.001	Falling
Semiárido	33.7	-3.6 (-6.7;-0.5)	0.027	Falling
Cerrados	41.8	-7.2 (-8.6;-5.8)	< 0.001	Falling
**Detection rate with** **G2D at the time of diagnosis (per 100,000 inhabitants)**	**2.0**	**-4.4 (-7.0;-1.8)**	**0.003**	**Falling**
**Proportion of new multibacillary cases (%)**	**58.4**	**4.1 (3.8;4** **.5)**	**< 0.001**	**Rising**
**Proportion of new paucibacillary cases (%)**	**41.6**	**-6** **.3 (-7.6;-4.9)**	**< 0.001**	**Falling**

a) Average detection rate: Average number of new cases per year of diagnosis, divided by the resident population in the middle year (2014), multiplied by 100,000 inhabitants, except for proportion of multibacillary and paucibacillary cases, for which we used the average number of cases according to operational classification; b) APC: Annual percentage change; c) 95%CI: 95% confidence interval; d) Wald test.

Figure 1B shows the spatial distribution of the average new leprosy case detection rate according to the health regions where the diagnosed people reside. From 2007 to 2011, the Chapada das Mangabeiras (45.1/100,000 inhabitants), Entre Rios (61.0/100,000 inhabitants), Carnaubais (64.5/100,000 inhabitants) and Vale dos Rios Piauí and Itaueiras (69.0/100,000 inhabitants) regions had a hyperendemic leprosy situation (rate greater than 40.0/100,000 inhabitants). In the second five-year period, only the Entre Rios (40.9/100,000 inhabitants) and the Vale dos Rios Piauí and Itaueiras (45.3/100,000 inhabitants) regions were considered to be hyperendemic. From 2017 to 2021, all regions had very high rates, except Vale do Sambito (17.6/100,000 inhabitants) and Cocais (18.4/100,000 inhabitants). Considering the average for the total period from 2007 to 2021, leprosy was hyperendemic in the Carnaubais (41.0/100,000 inhab.), Entre Rios (44.4/100,000 inhab.) and the Vale dos Rios Piauí and Itaueiras (46.7/100,000 inhab.) regions.

## DISCUSSION

We analyzed the epidemiological characteristics, the temporal trend and the spatial distribution of leprosy cases and indicators in Piauí over 15 years. There was a higher proportion of cases in males, those aged 40 to 59 years, those of mixed race/skin color, in the Entre Rios health region and in the Meio-Norte macro-region. Regarding clinical aspects, multibacillary cases, with grade 0 disability and the borderline form, were the most frequent.

Despite the downward trend in the overall detection rate, some regions of the state still showed very high values ​​for this indicator, compatible with hyperendemicity, according to Ministry of Health parameters.^8^ The same downward trend was seen for the new case detection rate according to sex, race skin/color and age group, regarding the rate of cases with grade 2 disability at diagnosis and the proportion of paucibacillary cases. The proportion of multibacillary cases was the only indicator with a rising trend.

The higher proportion and higher detection rate in males emphasize behavioral and cultural factors and the way in which health services are organized to meet the needs of this population. Furthermore, leprosy among men reflects patterns of disease, with a more severe clinical picture, greater occurrence of physical disabilities, lower cure rate, higher treatment abandonment rate, greater reporting of relapses and higher mortality.^10^

Predominance of leprosy in people of mixed and Black race/skin color, who also bear a historical legacy of discrimination and stigma, translates into unequal risks of acquiring the disease and social inequities.^11^ By being part of the social segments with the poorest living conditions, there is increased risk of acquiring leprosy and developing physical sequelae. In Piauí, one of the poorest Brazilian states, the reality of social vulnerability has already been highlighted as a determining factor in the increase in the number of cases.^5^

Higher detection rates in the Indigenous population date back to periods prior to slavery and colonization. Historically isolated, indigenous populations still suffer from difficulty in receiving medical care due to the distance of indigenous reserves from hospital centers and the logistics of health professionals traveling. There is also the language barrier and the culture shock with the Indigenous concept of health, with its traditional customs and rituals. As the development of quality health services depends on political decisions, unfortunately, traditional communities in Piauí continue to be neglected. However, in certain places there has already been significant progress in Indigenous health care with the strengthening of public care policies, such as the expansion of the *Mais* *Médicos* (More Doctors) Program.^12^

The heterogeneous pattern of the disease in different health regions reflects demographic, genetic, socioeconomic, environmental and cultural factors regarding occurrence of leprosy, these being factors that overlap geographically.^13^ Piauí is located in the area of climatic transition between the pre-Amazon and the semiarid, with different forest formations, with *Caatinga* (semiarid) standing out in 37% of the territory and the *Cerrado* (savanna) in 33%.^14^ Added to the demographic particularities of the state’s more than 200 municipalities,^7^ Piauí has significant socio-environmental heterogeneity, this being an important determinant of the health-disease process.

This geographic distribution pattern showed differences over the years, with changes in regions regarding high, very high endemicity and hyperendemicity situations. However, in all the time intervals analyzed there was always emphasis on the Vale dos Rios Piauí and Itaueiras, Entre Rios and Carnaubais regions. As Entre Rios covers the state capital and municipalities neighboring the state’s health reference centers and regional hospitals, this explains, in part, it having the largest number of case notifications.^6^ This contrasted with other regions of the state with poorer conditions, where limited access to health services led to leprosy being underdiagnosed.

Despite its falling trend, the average detection rate in children under 15 years of age reached hyperendemicity. This coefficient is used to monitor active transmission of the disease in the community, especially within families. Its reduction is therefore essential for leprosy control.^15^ Lack of information about the disease among the poorest populations and the lack of support groups and health professionals trained in achieving early diagnosis make adequate treatment difficult and facilitate transmission. There is also fear of seeking medical care due to marginalization and social stigma surrounding the disease.^16^

The rate of new cases with grade 2 disability at the time of diagnosis provides assessment of sequelae caused by leprosy. Its downward trend in the state of Piauí may hide the real situation of late diagnosis. This indicator remained higher than the rate for the Northeast region and for Brazil as a whole in the period from 2007 to 2021. It is essential to qualify and expand early detection, prevention, timely treatment and rehabilitation actions, especially among the most vulnerable populations. Due to the importance of monitoring and analyzing the epidemiological impact of the disease, reducing this rate is one of the priority goals of the National Strategy for Combating Leprosy (*Estratégia Nacional para o Enfrentamento da Hanseníase*).^17^

Late diagnosis and incorrect treatment can lead to polyneuropathy caused by leprosy, which compromises peripheral nerves with loss of muscle contraction capacity and skin lesions.^18^ As the disease affects people of economically active age, disability results in financial losses, especially when it is impossible to work, preventing them from remaining in jobs or occupations and causing problems with reintegration into the job market, marginalization in the production chain, psychological problems caused by social isolation and, therefore, considerable loss of quality of life. Consequently, there is an increase in public spending on medical care and social services for these people.^19^

Regarding the proportion of paucibacillary cases, the falling trend could indicate attenuation of the population’s exposure to the bacillus, thus signaling greater control of the endemic and less active transmission of the disease.^20^ However, the rising trend in the proportion of new multibacillary cases becomes more relevant, as this form of the disease is responsible for transmission and these individuals have large quantities of bacilli in the dermis and mucous membranes, being prone to spreading them in the environment. Once again, the increase in multibacillary indicates ineffective and/or late diagnosis of the disease.^21^

The higher proportion of the multibacillary form among the total number of people with leprosy demonstrates that there was a high circulation of Hansen’s bacillus in the state of Piauí during the study period. Due to the greater risk of complications in these people, it is essential to provide appropriate treatment to combat the transmission chain.^17^ Furthermore, some studies show that the pathophysiology of leprosy in this form has greater association with the development of reactional episodes, these being important causes of physical disability.^22^

Although the scope of this study has been little replicated in the scientific literature, similar studies have been carried out in other states. Results found for the state of Maranhão, in the 2001 to 2015 time series, also showed predominance of cases in males, aged between 35 and 64 years. However, the majority were of the paucibacillary operational classification and had grade 1 disability. There was also a falling trend for the overall detection rate and for 11 of the state’s 19 health regions, as well as for detection in children under 15 years of age. However, there was a significant rising trend in the rate of cases with grade 2 disability.^23^ A study in the state of Pernambuco, from 2011 to 2021, analyzed the spatial pattern of leprosy and found heterogeneity between municipalities in the overall detection rate and in those under 15 years of age, with values ​​ranging from low to hyperendemic.^24^

In the state of Bahia, as in Piauí, higher case frequencies were found in males and among people of mixed race/skin color. With rates considered very high, the pattern between different health regions was also uneven. The detection rate in children and the proportion of multibacillary cases showed the same trend as in Piauí, namely reduction and increase, respectively. However, the trend in the overall detection rate was stationary and there was an increase in cases with grade 2 disability. Furthermore, different trends were found for the indicators studied, with females and males being analyzed separately.^13^ Another study carried out in Bahia showed that factors related to higher detection rates include lower *per capita* income, a higher proportion of poor people in the municipality’s population and a greater number of people living together in the same household.^25^

Finally, this study included the years 2020 and 2021, coinciding with the context of the COVID-19 pandemic, a period of important changes in the global and regional health surveillance panorama. In Brazil, most activities related to NTDs were suspended, with consequent delays in diagnosis, treatment, morbidity management and disability prevention. One study showed a significant reduction in the detection of new leprosy cases, considering only the years of the pandemic, at a national level. In the same study, regarding the state of Piauí, a tendency towards a reduction in the rate was also found.^26^ Therefore, the decrease in the number of new cases during the pandemic should not be interpreted in isolation as a strengthening of the health system, but rather as shortcomings in diagnosis, with a greater impact on the socially vulnerable population.^4^

Potential limitations of our study refer to the origin of the data analyzed, obtained from secondary sources, which may contain inaccurate data due to underreporting and inadequate filling out of records and forms. Furthermore, as an ecological study, its conclusions at the aggregate level cannot be extrapolated to the individual level, which is a useful design for raising hypotheses to be confirmed by other types of study. 

However, this study was useful in demonstrating the persistence of leprosy as a highly prevalent disease in Piauí, despite the downward trend for most indicators. Detection rates are still at worrying levels, which place Piauí among the most endemic of the Brazilian states. This reality suggests shortcomings in surveillance and control actions, which makes it essential to strengthen actions to eliminate the disease, especially in the most affected regions. Furthermore, in the context of the COVID-19 pandemic, the reduction in most indicators may be due to the interruption of prevention, diagnosis and treatment actions during the period of restricted access to health services during social distancing recommendations and interruption of some types of primary health care services.

This study enables a greater understanding of the epidemiological situation of leprosy in Piauí and, consequently, supports the taking of action by local health authorities, with regard to carrying out measures aimed at early diagnosis, ensuring the start of treatment and adherence, as well as the prevention and reduction of physical disabilities related to leprosy, in a scenario that is even more challenging in the wake of the COVID-19 health emergency.
